# Supplementary material for: Early Notice Pointer, an IoT-like Platform for Point-of-Care Feet and Body Balance Screening
Source: Micromachines (Basel). 2022 Apr 27;13(5):682. doi: 10.3390/mi13050682 (PMC9144081; doi:10.3390/mi13050682)
Supplement: Supplementary file 1 [file micromachines-13-00682-s001.zip › micromachines-1658284-SI.pdf]

**Supplementary Materials**

**Figure S1:** The workflow of ENP to explain the feedback loops for measurement consistency

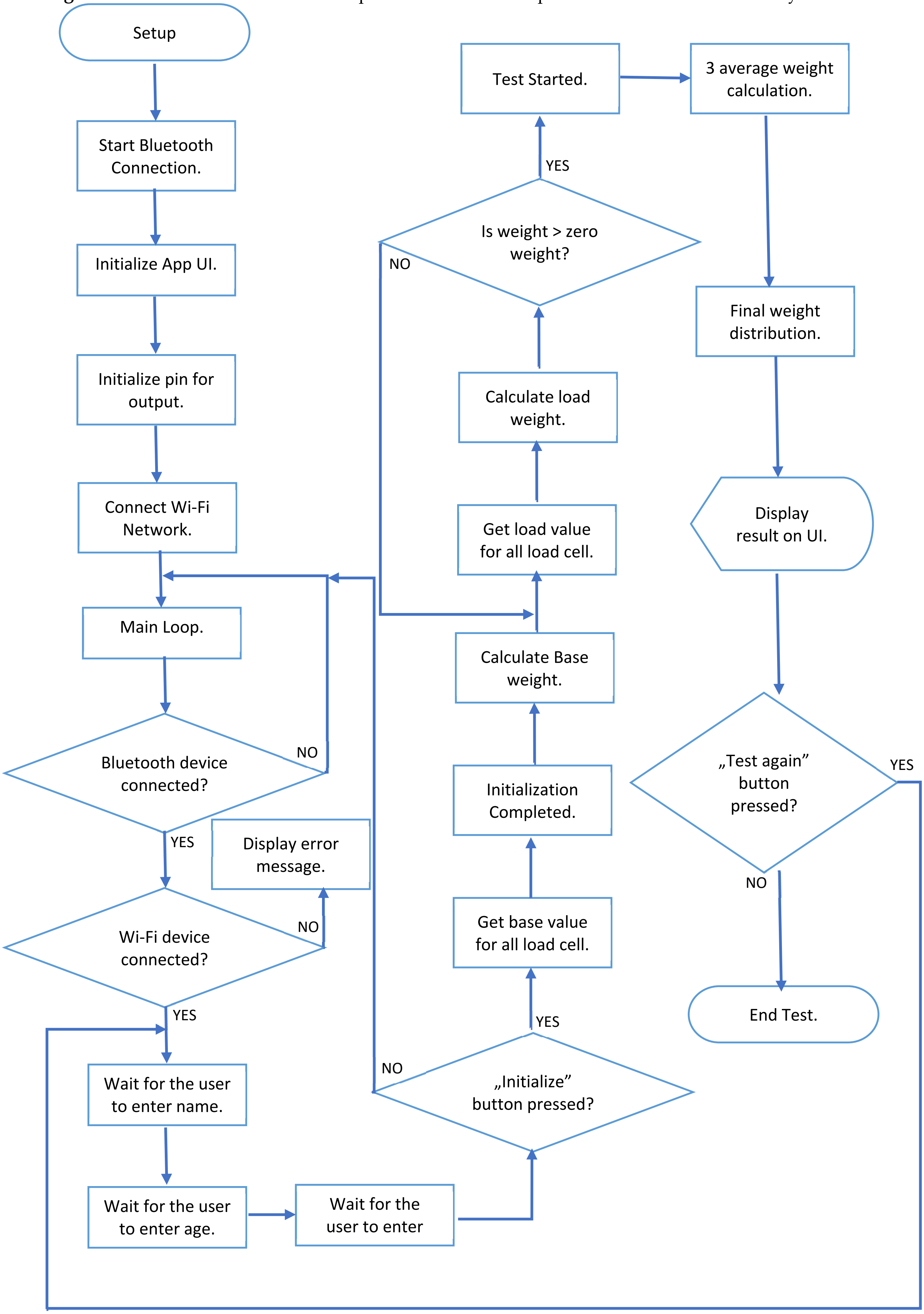

**Figure S2:** The workflow of ENP to explain the feedback loops to users

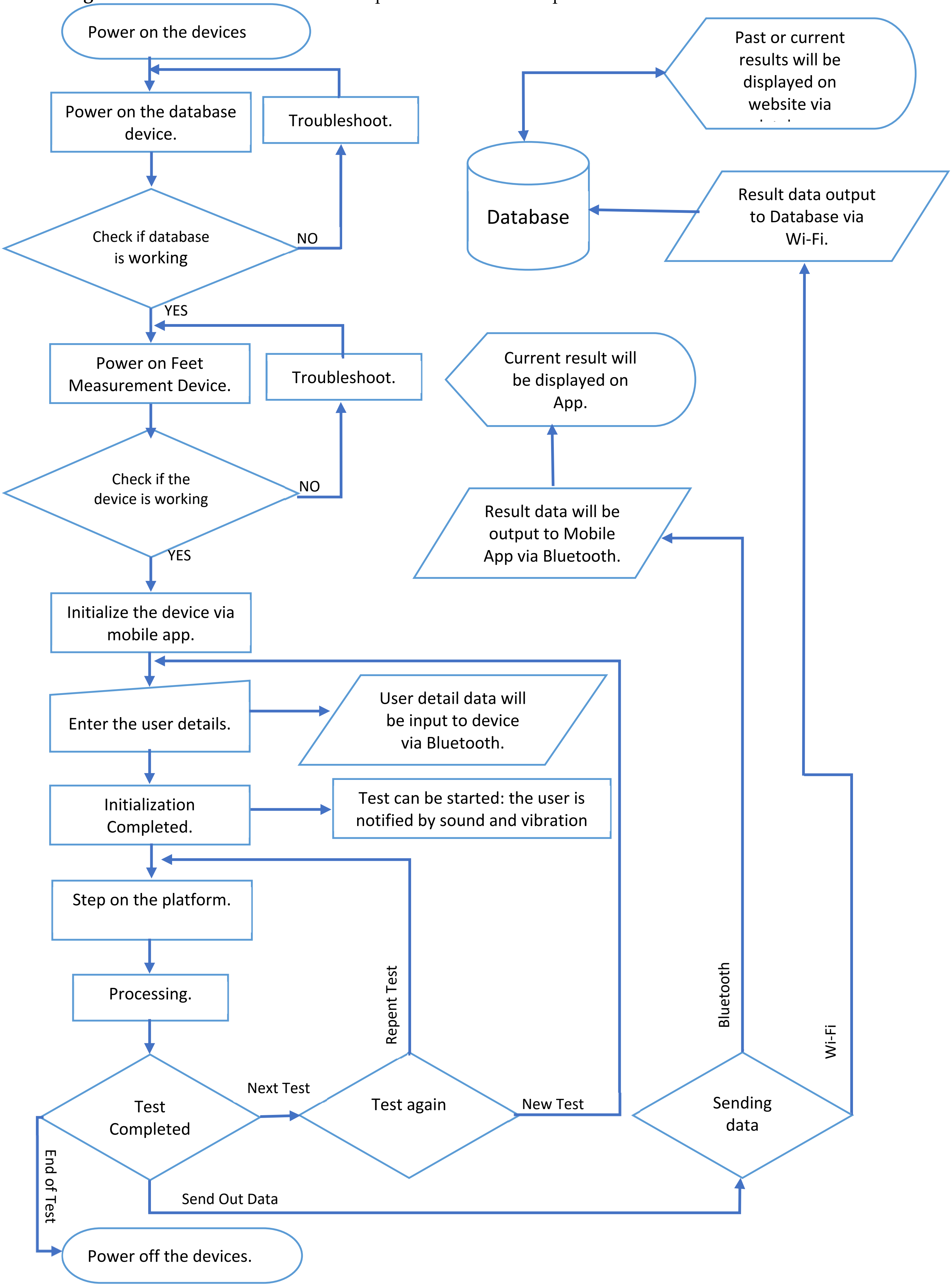

**Table S1:** The T-test results to indicate differences between the data acquired with the prototype and the foot scanners.

| Test Matscan vs. Prototype | Left | Right | Left forefoot | Left rearfoot | Right forefoot | Right rearfoot |
|---|---|---|---|---|---|---|
| T-Test (test1&2) 2tail type1 | 7.00E-32 | 9.96E-31 | 2.00E-15 | 5.81E-16 | 6.60E-12 | 2.00E-15 |
| T-Test (test1&2) 2tail type2 | 6.00E-50 | 1.32E-47 | 2.00E-23 | 1.29E-21 | 1.50E-15 | 2.02E-23 |
| T-Test (test1&2) 2tail type3 | 3.00E-47 | 3.39E-45 | 5.00E-18 | 6.32E-19 | 2.00E-12 | 7.32E-19 |
| RS FootScan vs. Prototype | | | | | | |
| T-Test (test1&2) 2tail type1 | 3.38E-01 | 3.36E-01 | 1.25E-01 | 3.44E-01 | 1.15E-11 | 1.87E-09 |
| T-Test (test1&2) 2tail type2 | 3.59E-01 | 3.57E-01 | 1.77E-01 | 3.60E-01 | 4.98E-09 | 5.62E-07 |
| T-Test (test1&2) 2tail type3 | 3.59E-01 | 3.57E-01 | 1.77E-01 | 3.60E-01 | 5.02E-09 | 5.57E-07 |
